# Supplementary material for: Moesin integrates cortical and lamellar actin networks during Drosophila macrophage migration
Source: Nat Commun. 2025 Feb 6;16:1414. doi: 10.1038/s41467-024-55510-5 (PMC11802916; doi:10.1038/s41467-024-55510-5)
Supplement: Supplementary file 1 — Supplementary Information [file 41467_2024_55510_MOESM1_ESM.pdf]

Supplementary Information

Moesin integrates cortical and lamellar actin networks during *Drosophila* macrophage migration

Authors: Besaiz J. Sánchez-Sánchez[1], Stefania Marcotti[1], David Salvador-Garcia[1], María-del-Carmen Díaz-de-la-Loza[1], Mubarik Burki[1], Andrew J. Davidson[2], Will Wood[3], Brian M. Stramer[1*]

Affiliations:

[1]Randall Centre for Cell and Molecular Biophysics, King's College London, SE1 1UL London, UK.

[2]Wolfson Wohl Cancer Research Centre, School of Cancer Sciences, University of Glasgow, Garscube Estate, Switchback Road, Bearsden, G61 1BD, Glasgow, UK.

[3]Centre for Inflammation Research, Institute for Regeneration and Repair, University of Edinburgh, 5 Little France Drive, Edinburgh Bioquarter, EH16 4UU, Edinburgh, UK.

[*] E-mail: brian.m.stramer@kcl.ac.uk

Supplementary Figures

Supplementary Fig. 1

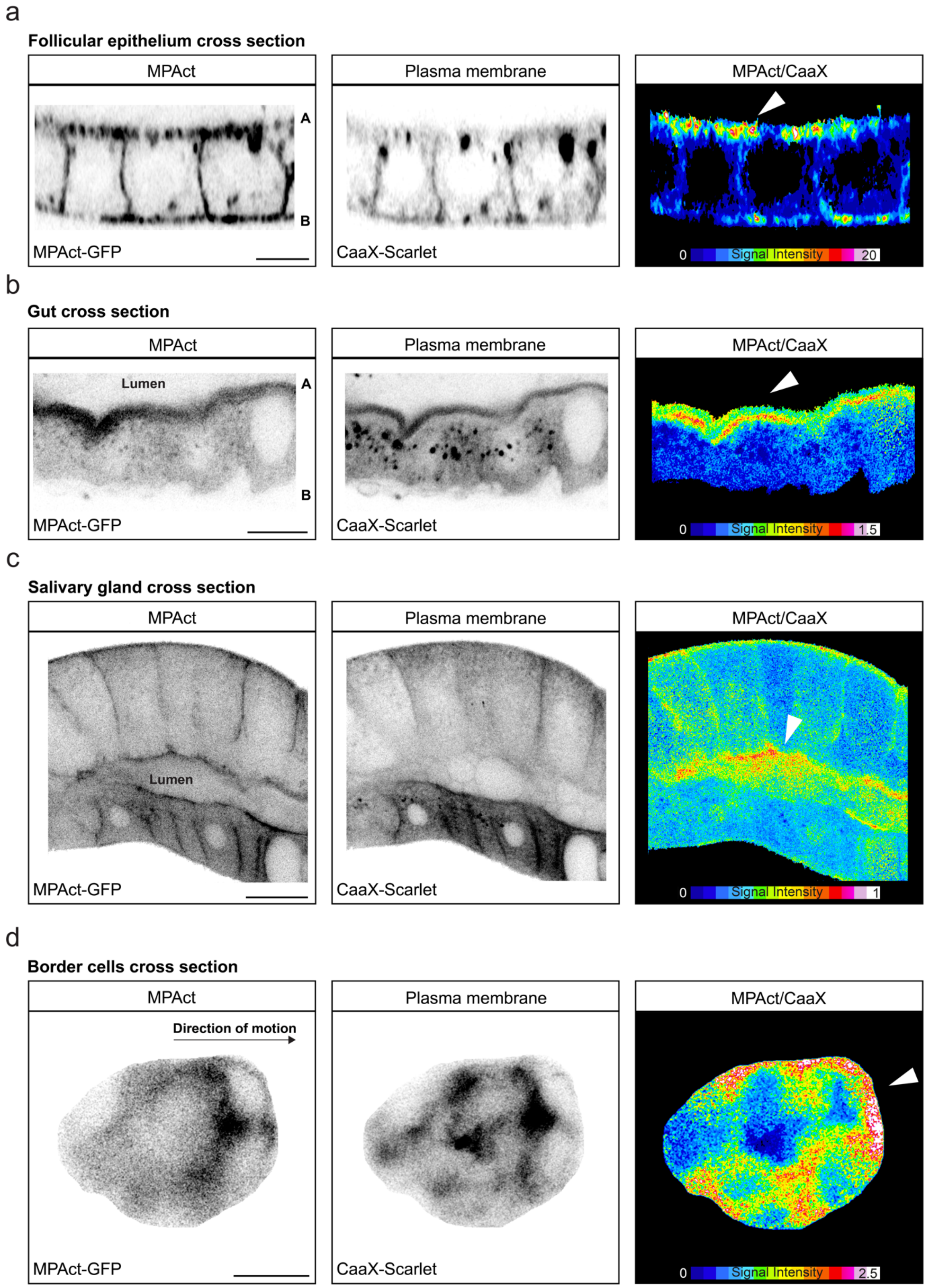

Supplementary Fig. 1: Ratiometric analysis of MPAct highlights the actin cortex in several *Drosophila* tissues

a, Ratiometric imaging of a membrane-proximal actin probe (MPAct) with membrane probe (CaaX) in the follicular epithelium (A and B highlight apical and basal sides, respectively). Note an increase in membrane-associated actin at the apical side (arrowheads). Scale bar, 5 µm. b, Ratiometric imaging of MPAct with CaaX in the gut epithelium (A and B highlight apical and basal sides, respectively). Note an increase in membrane-associated actin at the apical side of the gut cells (arrowheads). Scale bar 10 µm. c, Ratiometric imaging of MPAct with CaaX, in the salivary gland epithelium. Note an increase in membrane-associated actin at the apical side of the epithelium facing the lumen (arrowheads). Scale bar 50 µm. d, Ratiometric imaging of MPAct with CaaX during border cell migration, highlighting an increase in membrane-associated actin around the border cell cluster (arrowheads). Scale bar 10 µm.

Supplementary Fig. 2

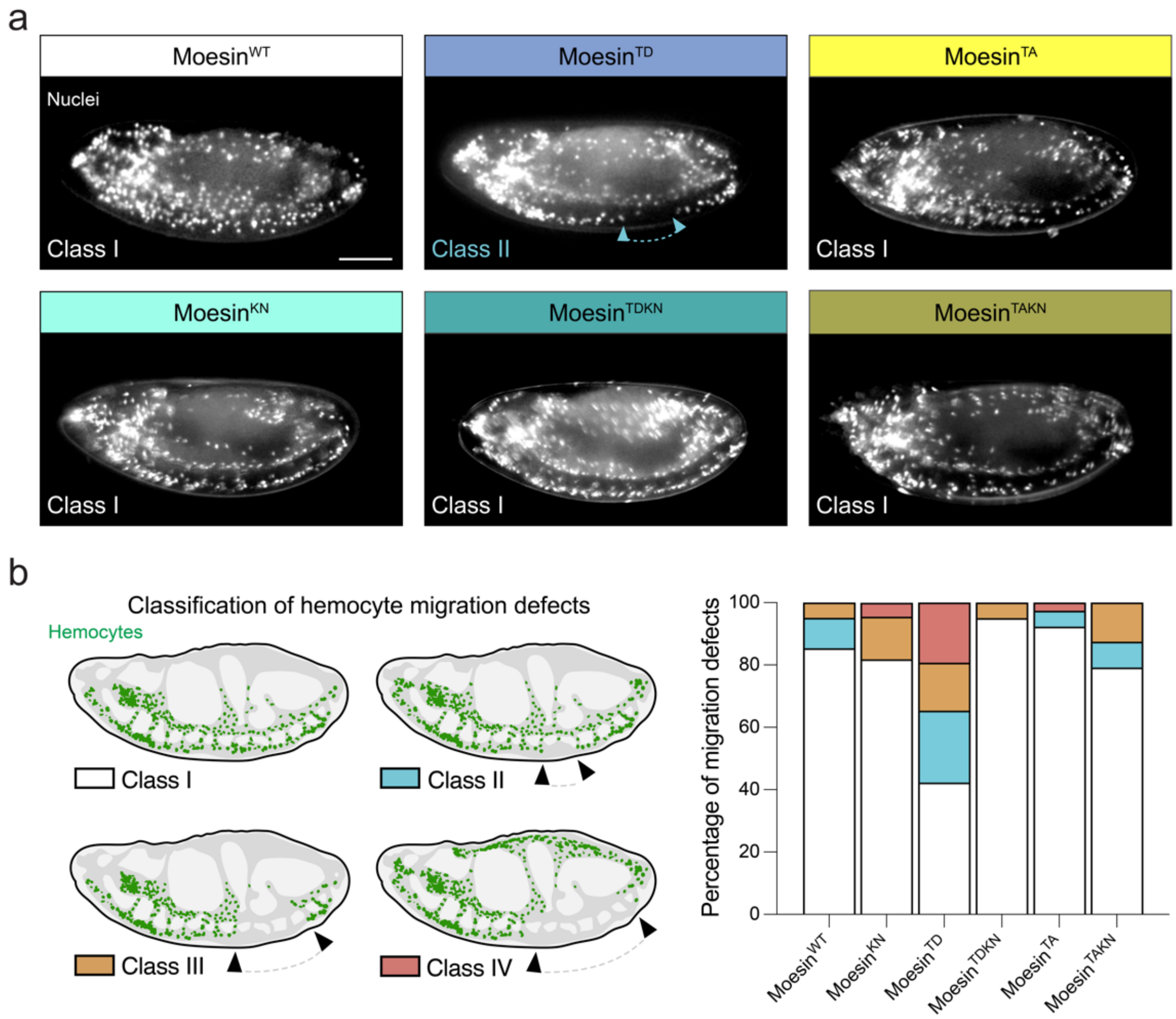

Supplementary Fig. 2: Expression of a single copy of Moesin transgenes reveals mild motility defects

a, Analysis of hemocyte migration when single copies of the transgenic Moesin constructs are driven specifically in hemocytes, revealing mild dispersal defects. Scale bar, 100 µm. b, (left panel) Schematic highlighting the classification of the degree of hemocyte developmental dispersal defects (as shown in Figure 3e). (right panel) Quantification of the degree of developmental dispersal defects when single copies of the transgenic Moesin constructs are driven specifically in hemocytes (n = 41 $Moesin^{WT}$, 22 $Moesin^{KN}$, 26 $Moesin^{TD}$, 20 $Moesin^{TDKN}$, 39 $Moesin^{TA}$ and 24 $Moesin^{TAKN}$ embryos).

Supplementary Fig. 3

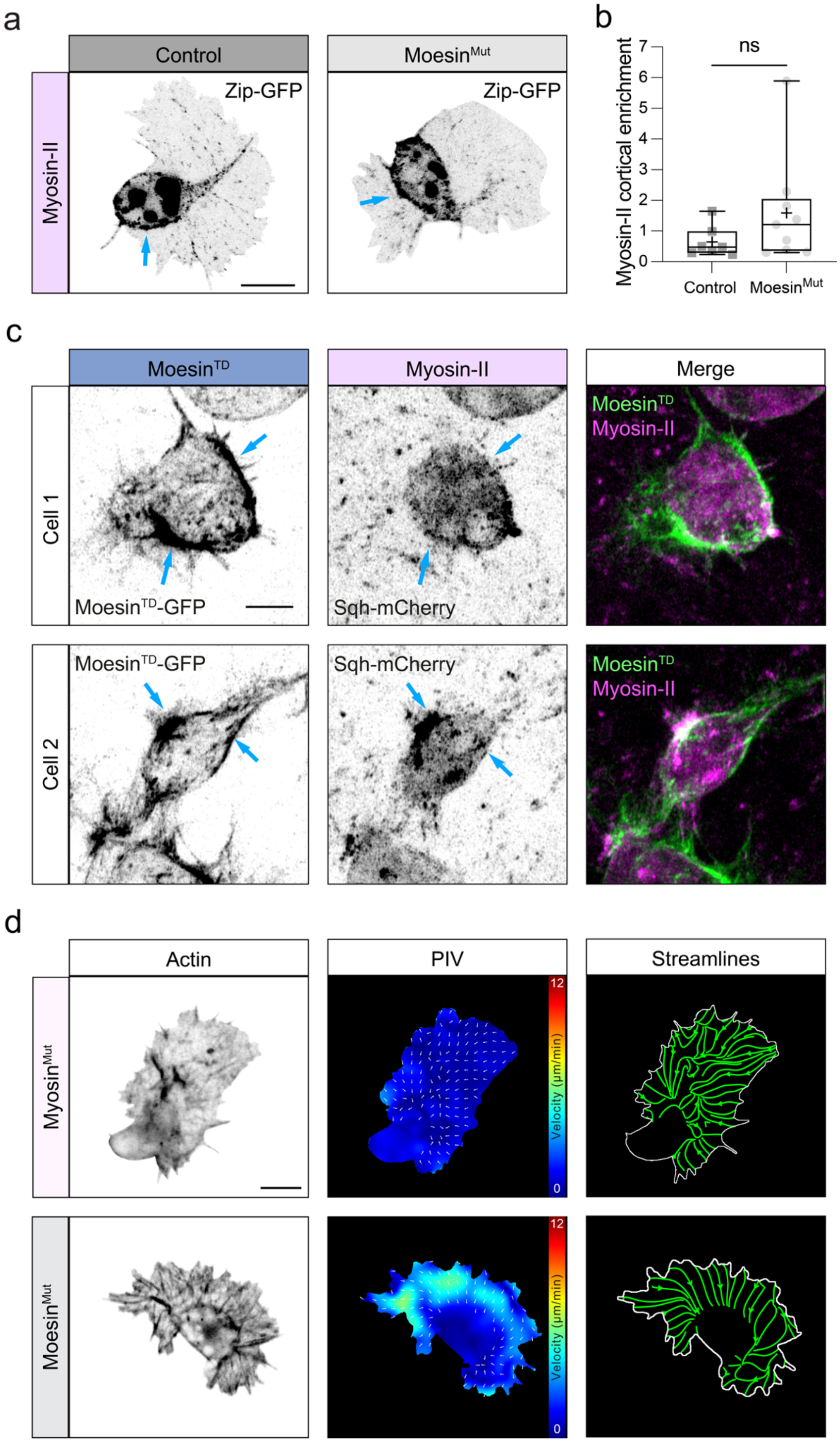

Supplementary Fig. 3: Moesin and Myosin-II have distinct cortical functions in hemocytes

a, Control and $Moesin^{Mut}$ macrophages expressing Myosin-II showing enrichment at the cell cortex (arrows). b, Quantification of Myosin-II enrichment at the cell cortex showing no statistical difference in the absence of Moesin. $^{ns}P = 0.1738$. Mann–Whitney two-tailed tests. Boxplot shows medians, 25th and 75th percentiles as box limits, minimum and maximum values as whiskers; each datapoint is displayed as a marker (n = 7 Control and 9 $Moesin^{Mut}$ hemocytes). c, Examples of cells co-expressing a single copy of $Moesin^{TD}$ and Myosin-II at the cortex (arrows). Note that $Moesin^{TD}$ has a more widespread localization surrounding the cortex compared to Myosin. d, Actin distribution, PIV analysis, and streamline representation of actin flow in $Myosin^{Mut}$ and $Moesin^{Mut}$ hemocytes. Actin flow organization is perturbed in $Myosin^{Mut}$ cells, however, $Moesin^{Mut}$ hemocytes show a coherent flow field, suggesting that these two proteins are playing relatively distinct roles in the lamella. Scale bars 10 µm.
