# Supplementary material for: Moesin integrates cortical and lamellar actin networks during Drosophila macrophage migration
Source: Nat Commun. 2025 Feb 6;16:1414. doi: 10.1038/s41467-024-55510-5 (PMC11802916; doi:10.1038/s41467-024-55510-5)
Supplement: Supplementary file 2 — Description Of Additional Supplementary File [file 41467_2024_55510_MOESM2_ESM.pdf]

Description of Additional supplementary files

Supplementary Movie 1

Time-lapse movie of a hemocyte expressing LifeAct-GFP with an overlay of a deformation map of the actin network, which was calculated from particle image velocimetry (PIV) analysis of the actin flow. This analysis highlights enhanced actin network deformation at the boundary between the lamella and cell body. Related to Figure 1a.

Supplementary Movie 2

Time-lapse movie of a hemocyte expressing LifeAct-GFP (left panel) and the divergence in the actin flow, which was calculated from PIV analysis (right panel). In this movie only negative divergence is highlighted, which reveals regions of actin network compression. Note the increase in actin network compression within the region surrounding the cell body. Related to Figure 1b.

Supplementary Movie 3

3D reconstruction of a hemocyte expressing LifeAct-GFP, highlighting the transition between the flat lamellar network and the rounded cell body. Additionally, note the accumulation of actin at the boundary of the lamella with the cell body. Related to Figure 1c.

Supplementary Movie 4

3D reconstruction of hemocytes expressing MoesinWT -GFP (left panel) or PIP2 (PLC-PHGFP) (right panel). This 3D analysis reveals an increase in these actin cortex regulators around the cell body. Related to Figure 1d.

Supplementary Movie 5

3D reconstruction of hemocytes expressing the membrane-proximal actin probe (MPAct). (top panel) Images of a hemocyte co-expressing MPAct-GFP and an actin probe (F-tractin-Scarlet) along with a ratiometric measurement of MPAct/Ftractin. (bottom panel) Images of a hemocyte co-expressing MPAct-GFP and a plasma membrane probe (CaaX-Scarlet) along with a ratiometric measurement of MPAct/CaaX. Note the enrichment of the MPAct signal surrounding the hemocyte cell body. Color scale in the ratiometric image: black (low level) to white (high level). Related to Figure 1e.

Supplementary Movie 6

Time-lapse movie of hemocytes expressing the membrane-proximal actin probes as analyzed in Supplementary Video 5 during their random developmental migration. Note the pulsatile MPAct signal surrounding the cell body suggesting a dynamic actin cortex during motility. Related to Figure 1e.

Supplementary Movie 7

3D reconstruction of a Control (top panel) and MoesinMut (bottom panel) hemocyte co-expressing MPAct-GFP and a plasma membrane probe (CaaX-Scarlet) along with a ratiometric measurement of MPAct/CaaX. This analysis reveals low levels of MPAct signal around the cell body in MoesinMut. Additionally, note that in the absence of Moesin there is a flattening of the cell body. Color scale in the ratiometric image: black (low level) to white (high level). Related to Figure 2b.

Supplementary Movie 8

Time-lapse movie of Control (top panel) and MoesinMut (bottom panel) hemocytes during their random developmental dispersal co-expressing LifeAct-GFP and a nuclei label (RedStinger). Note the altered distribution of MoesinMut hemocytes along with frequent disruption of cell polarity. Related to Figure 3a.

Supplementary Movie 9

3D reconstruction of hemocytes transgenically expressing two copies of MoesinWT (top panel) or two copies of MoesinTD (bottom panel) while also coexpressing MPAct-GFP and a plasma membrane probe (CaaX-Scarlet). Note that in hemocytes expressing MoesinTD the ratiometric measurement of MPAct/CaaX highlights an altered MPAct distribution, with an increase in signal within the lamella. Color scale in the ratiometric image: black (low level) to white (high level). Related to Figure 4e.

Supplementary Movie 10

Time-lapse movie of hemocytes during their random developmental dispersal transgenically expressing a single copy of GFP-tagged MoesinTD while also labelling the actin network (srpHemoMoe::3xmCherry). Note the flow of MoesinTD within the lamella towards the cell body and the pulsatile dynamics surrounding the cortex. Related to Figure 5.

Supplementary Movie 11

Time-lapse movie of Control (left panel) and MoesinMut (right panel) hemocytes expressing LifeAct-GFP with an overlay of the morphodynamic analysis. The magenta arrows highlights the instantaneous direction of motion and the black contour reveals the portion of the lamella correlated with cell motion. Note the fluctuating polarity and movement of the MoesinMut hemocyte, which is consistent with their reduction in cell persistence. Related to Figure 7.
